# Enhancing drought tolerance in *Pisum sativum* and *Vicia faba* through interspecific interactions with a mixed inoculum of *Rhizobium laguerreae* and non-host beneficial rhizobacteria

**DOI:** 10.3389/fpls.2025.1528923

**Published:** 2025-02-26

**Authors:** Asma Hami, Imane El Attar, Najoua Mghazli, Salah Ennajeh, El Houcine Ait-Ouakrim, Meryeme Bennis, Said Oulghazi, Bouabid Badaoui, Jamal Aurag, Laila Sbabou, Kaoutar Taha

**Affiliations:** ^1^ Microbiology and Molecular Biology Team, of Plant and Microbial Biotechnology, Biodiversity and Environment, Faculty of Sciences, Mohammed V University, Rabat, Morocco; ^2^ AgroBioscience Program, University Mohammed VI Polytechnic (UM6P), Benguerir, Morocco; ^3^ Institut de Recherche en Mines et Environnement, Université du Québec en Abitibi-Témiscamingue, Rouyn-Noranda, QC, Canada; ^4^ Biodiversity, Ecology and Genome Laboratory of Zoology and General Biology, Center of Plant and Microbial Biotechnology, Biodiversity and Environment, Faculty of Sciences, Mohammed V University, Rabat, Morocco; ^5^ African Sustainable Agriculture Research Institute (ASARI), Mohammed VI Polytechnic University (UM6P), Laayoune, Morocco

**Keywords:** *Rhizobium laguerreae*, PGPR, *Pisum sativum*, *Vicia faba*, legumes, drought stress

## Abstract

**Introduction:**

Harnessing plant growth-promoting rhizobia presents a sustainable and cost-effective method to enhance crop performance, particularly under drought stress. This study evaluates the variability of plant growth-promoting (PGP) traits among three strains of *Rhizobium laguerreae* LMR575, LMR571, and LMR655, and two native PGP strains *Bacillus* LMR698 and *Enterobacter aerogenes* LMR696. The primary objective was to assess the host range specificity of these strains and their effectiveness in improving drought tolerance in three legume species: *Pisum sativum*, *Vicia faba*, and *Phaseolus vulgaris*.

**Methods:**

*In-vitro* experiments were conducted to assess the PGP traits of the selected strains, including phosphate solubilization, indole-3-acetic acid (IAA) production, and siderophore production. Greenhouse trials were also performed using a mixed inoculum of performing strains to evaluate their effects on plant physiological and biochemical traits under drought conditions.

**Results:**

Significant variability in PGP traits was observed among the strains. *R. laguerreae* LMR655 exhibited the highest phosphate solubilization (113.85 mg mL^-1^ PO_4_^2-^), while *R. laguerreae* LMR571 produced the highest IAA concentration (25.37 mg mL^-1^). *E. aerogenes* LMR696 demonstrated 82% siderophore production. Symbiotic interactions varied, with *R. laguerreae* LMR571 and LMR655 forming associations with *P. sativum* and *V. faba*, but none establishing compatibility with *P. vulgaris*. Greenhouse experiments showed that a mixed inoculum of *R. laguerreae* LMR571, LMR655, and *E. aerogenes* LMR696 significantly improved proline, total soluble sugars, proteins, and chlorophyll content under drought stress, with *V. faba* showing the strongest response.

**Discussion:**

These findings highlight the importance of strain selection based on host specificity and PGP potential. The enhanced drought tolerance observed suggests that tailored microbial inoculants can improve legume resilience in water-limited environments. This study provides valuable insights for optimizing bioinoculant formulations to enhance crop performance under drought stress.

## 1 Introduction

Climate change is a pressing global challenge that drives environmental crises, impacting ecosystems, biodiversity, human health, and food security (Abbass et al., 2022). Over the past 65 years, both natural and human-induced factors, including industrial activities and agriculture, have intensified its effects (Wu et al., 2020; Abbass et al., 2022). The agricultural sector, accounting for 30-40% of greenhouse gas (GHG) emissions, significantly contributes to global warming through practices such as excessive use of chemical fertilizers, mechanization, and fossil fuel consumption (Tubiello et al., 2021).

Climate change also disrupts hydrological cycles, leading to erratic rainfall, shrinking water bodies, and frequent droughts, which severely impact crop productivity, especially in water-scarce regions like Morocco. Situated between North Africa’s arid zone and Europe’s temperate climate, Morocco faces rising temperatures, decreasing rainfall, and increasing droughts, which threaten agricultural sustainability (Simonneaux et al., 2015; Woillez, 2019). Drought stress, in particular, hampers legume production in arid and semi-arid regions (Jagadish et al., 2016). Leguminous crops, vital for human nutrition and soil health, are especially vulnerable to such conditions. These crops form symbiotic relationships with N2-fixing bacteria, such as rhizobia, which enhance biological N2 fixation and improve soil fertility (Sprent et al., 2017).

The application of rhizobia as biofertilizers has proven effective in improving N2 fixation and soil fertility, particularly under stress conditions. Recent studies suggest that co-inoculation with native rhizobia and plant growth-promoting rhizobacteria (PGPR) significantly enhances drought tolerance in legumes. Notably, *Rhizobium laguerreae* has demonstrated remarkable potential in promoting plant growth under osmotic stress. Isolated from various leguminous crops across different countries (Saïdi et al., 2014; Taha et al., 2018), this species has shown beneficial effects on lentil growth under drought conditions when co-inoculated with native *Bacillus* sp. and *Enterobacter aerogenes* (Taha et al., 2022). Additionally, *R. laguerreae* has been shown to enhance productivity and phenolic composition in lettuce under saline conditions (Ayuso-Calles et al., 2020). These findings highlight the potential of *R. laguerreae* as a biofertilizer to enhance legume resilience and productivity in challenging environmental conditions.

The objective of this study is to evaluate the drought tolerance of three *R. laguerreae* strains, previously isolated from Lens culinaris root nodules (Taha et al., 2018), under controlled osmotic stress conditions. Specifically, the study aims to: a) Characterize the plant growth-promoting (PGP) traits of these strains, including auxin production, phosphate solubilization, and siderophore production, b) Assess their host range specificity with *Pisum sativus, Phaseolus vulgaris*, and *Vicia faba*, and c) Investigate their potential to form effective microbial mixed inoculum with other PGP rhizobacteria, such as *Bacillus* sp. and *Enterobacter aerogenes*, and evaluate their impact on drought resistance and legume productivity in water-limited environments.

## 2 Materials and methods

### 2.1 Bacterial strains

Three strains of *Rhizobium laguerreae* LMR 575, LMR 597, and LMR 655, along with two rhizosphere bacteria, *Bacillus* sp. LMR 698 and *Enterobacter aerogenesaer*. LMR 696 were used in this study. These strains were isolated from lentil (*Lens culinaris* Medik) root nodules and rhizosphere, respectively, grown in various regions in Morocco (Taha et al., 2018). They were selected based on their performance in improving lentil growth, in single or combined inoculations, under drought stress conditions, as well as their ability to withstand osmotic stress *in-vitro* using PEG_6000_ as a stressor agent (Taha et al., 2022).

### 2.2 Quantitative assessment of plant-growth-promoting traits under osmotic stress

#### 2.2.1 Indole-3-acetic-acid production

Auxin production of the selected strains was determined following Sarwar method (Sarwar et al., 1992). Freshly prepared bacterial cultures were inoculated in 15 ml of YEM medium amended with 50 μg mL^-1^ of L-tryptophane as a precursor for auxin production. Different concentrations of PEG_6000_ (5%, 10%, 20%, 30%, 35%) were added to induce osmotic stress. Bacterial cultures were then incubated at 28 ± 2 °C for three days at a shaking speed of 120 rpm. Two milliliters of bacterial cultures were collected every 24 hours to measure bacterial growth and auxin production. The amount of IAA production in each isolate was measured using the Salkowski reagent (Ehmann, 1977). After incubation, the cultures were centrifuged at 8000 rpm for 15 min at 4 °C, and the supernatant was mixed with Salkowski reagent then incubated in the dark for 30 min for color development. Absorbance was measured at 530 nm and the IAA concentration was estimated by using a standard IAA curve with pure IAA concentrations ranging from 0 to 100 µg mL^-1^ (Gordon and Weber, 1951).

#### 2.2.2 Siderophore production

The strains’ capacity to produce siderophores was tested using the CAS-shuttle assay method developed by Schwyn and Neilands (1987). Bacterial cultures were grown in iron-deficient minimal medium (El Attar et al., 2019), supplemented with the same PEG_600_ concentrations as mentioned above. After seven days of incubation, the bacterial cultures were centrifuged to remove the pellet. An equal volume of supernatant and CAS reagent was then mixed, and the optical absorption at 630 nm was measured. Siderophore production was estimated using the formula of Payne (1993).

#### 2.2.3 Inorganic phosphate solubilization under drought conditions

The inorganic phosphate (P) solubilization capacity was determined by using Pikovskaya’s medium (PVK) (Pikovskaya, 1948) supplemented with 0.05 g of Moroccan rock phosphate as a sole source of P. PEG_6000_ was added to the medium using the same concentrations mentioned above. Bacterial cultures were incubated at 28 °C for 3 days with shaking. After incubation, the cultures were centrifuged at 12 000 rpm for 15 min to collect the supernatant. Soluble P content in the supernatant was estimated following the Vanadate-molybdate method (Tandon et al., 1968). Absorbance was measured at 405 nm and soluble phosphorus in the medium was calculated from the standard curve of P concentration using KH_2_PO_4_.

### 2.3 Host range of *R. laguerreae* strains

#### 2.3.1 Seed germination

To determine the host range of *R. laguerreae* strains, four grain legume hosts were used: Lentil (*Lens culinars*), Pea (*Pisum sativum*), faba bean (*Vicia faba*), and common bean (*Phaseolus vulgaris*). The seeds were kindly provided by INRA (Institut National de Recherche pour l’Agriculture) Institute of Rabat, Morocco.

Seeds of *P. sativum*, *V. faba*, and *Phaseolus vulgaris* were surface sterilized by immersion in 70% ethanol for 2 minutes, followed by thorough rinsing with sterile distilled water to remove any ethanol residues. The seeds were then immersed in a 20% sodium hypochlorite solution for 20 minutes and rinsed again with sterile distilled water to eliminate any remaining traces of salt. Lentil seed sterilization was done following the same protocol described by Taha et al. (2018). After surface sterilization, all seeds were germinated in 0.7% agar plates at 28 °C in the dark for 2 days.

#### 2.3.2 Inoculation and experiment design

Five germinated seeds were transferred to plastic pots containing a mixture of an equal quantities of sterilized sand and surface-applied beads (Haweison and dilworth, 2016), then thinned to 3 after successful seedling establishment.

*R. laguerreae* strains were grown in liquid YEM medium at 28°C for 24h and bacterial density was standardized to 10^8^ CFU mL^-1^. Seedlings in each pot were inoculated with 1 mL of rhizobia culture.

Two sets of control plants were settled: non-inoculated non-fertilized seedlings (Control N0) and non-inoculated fertilized seedling supplied with mineral nitrogen (0.5 g L^–1^ KNO_3_) (Control N+). The experiment was carried out with three replicates using a Randomized Complete Block (RCB) design and all pots were watered twice a week with an N-free nutrient solution.

### 2.4 Assessment of plant growth improvement by bacterial mixed inoculation under drought stress

Based on the results of host range experiment and the evaluation of PGP traits under osmotic stress, *R. laguerreae* LMR 597 and LMR 655 and *E. aerogenes* LMR696 strains were selected based on their performance. The three strains were used as a mixed inoculum to further evaluate their effect of plant growth and development under water deficiency stress. The leguminous plants *P. sativum* and *V. faba* that had established a symbiosis with *R. laguerreae* strains were used in this experiment.

#### 2.4.1 *In-vitro* compatibility test

To ensure compatibility between rhizobial and PGPR strains intended for mixed inoculation, potential antagonistic interactions were assessed *in-vitro* following the procedure outlined by Mikiciński et al. (2016). The strains were co-cultured on nutrient agar plates, and zones of inhibition were monitored to detect antagonistic effects. Experiments were performed in triplicate to ensure reproducibility.

#### 2.4.2 Experiment design and layout

The soil used in this experiment was non-sterilized agricultural soil collected from a local site. The soil’s pH was 7.64 ± 0.05, the cation exchange capacity (CEC) was found to be 0.21 ± 0.02 mS/cm, the potassium content was 120.02 ± 12.50 ppm, while phosphorus was present at 58.45 ± 0.71 ppm. Nitrogen was measured at 0.038 ± 0.004%, and the organic matter content was 2.88 ± 0.91%. The dry matter content was 91.13 ± 0.26%, and magnesium was present at 7.25 ± 0.04%. The carbon content in the soil was measured at 1.81 ± 0.005%, with sodium present at 1.29 ± 0.06 m eq 100 g^-1^.

A preliminary irrigation test was conducted to determine the soil’s maximum water retention capacity, from which 30% and 70% moisture levels were selected to represent drought stress and well-watered conditions, respectively. The experimental setup included a total of 72 pots, randomly distributed across three blocks, with six replicates per treatment. The six treatments comprised: inoculated plants under drought stress, inoculated plants with normal irrigation, non-inoculated plants under drought stress, non-inoculated plants with normal irrigation, and two non-inoculated controls, negative (N0) and positive (N+), that was supplemented with N_2_ fertilizer (0.5 g L^-1^ KNO_3-_). Both were subjected to the same irrigation regime. Non-inoculated controls received no microbial treatment, while inoculated plants were treated with the bacterial mixed inoculum.

The plants were initially watered to meet their growth requirements, and drought stress was applied starting in the third week of growth, continuing until the flowering stage. After 8 weeks of growth, all plants were harvested for further analysis of growth parameters, including root and shoot biomass, chlorophyll content, and drought tolerance indicators.

The multi-strain mixed inoculum was prepared by combining equal proportions of fresh cultures of each strain, which were separately grown in liquid YEM medium. The mixed inoculum suspension was then adjusted to a final concentration of 10^8^ cfu mL^–1^ and applied immediately after transplantation, with a follow-up application after 15 weeks. The experiment was conducted following the RCB design, with six replicates per treatment.

#### 2.4.3 Plants’ physio-morphological characteristics

Six weeks into the experiment, the plants were harvested, and the measurements of nodule number, shoot, and root system lengths were measured. Plants shoot and root parts were oven-dried at 70°C for 48 hours and dry weights were recorded. For chlorophyll (a), (b), and carotenoid measurements, leaf segments of each treatment were immersed in 4 ml of 80% v/v acetone solution and incubated for 48 hours at 4°C (Amine-Khodja et al., 2022). Photosynthetic pigments were quantified according to Lichtenthaler and Wellburn’s (1983) equation.

#### 2.4.4 Biochemical characteristics

The proline content of all tested plants was extracted by using a cold extraction procedure by mixing plant samples with ethanol and left to incubate overnight at 4°C, then they were centrifuged for 5 min at 8000 rpm to retrieve the pellet and optical density of the supernatant was measured at 520 nm (Gibon et al., 2000). The detection of proline was related to a standard curve prepared as described by Carillo et al. (2011).

Anthocyanin biosynthesis was measured following the protocol of Mustilli et al., 1999. Fresh shoot biomass was incubated in a 1% HCl methanol solution in the dark for two nights at +4°C. After incubation, chloroform and distilled water were added to the supernatant retrieved by centrifugation. The absorbance of the supernatant was measured at 535 and 650 nm, using the extraction solution as the blank. Anthocyanin content was calculated as the difference in absorbance (A535 - A650) per mg of fresh material.

Total soluble sugar content was estimated using the method described by DuBois et al. (1951). Plant material was homogenized with 80% ethanol and incubated for two nights. After centrifugation, the collected supernatant was added to a reaction mixture containing 5% phenol and 96% sulfuric acid. The mixture was then incubated at 30°C for 20 minutes. The optical density of the resulting solution was measured at 490 nm, and the total soluble sugar content was estimated per gram of fresh weight, referring to a standard curve prepared with varying concentrations of glucose.

Protein production was calculated by grinding fresh leaves with a cooled phosphate buffer (pH=7.8). The supernatant obtained after centrifugation and pellet removal was incubated with Bradford reactant at 37°C for 5 min. The mixture absorbance was measured at 595 nm after incubation, and the concentration of soluble protein was assessed by using the Bovine serum albumin standard curve following the method described by Bradford (1976).

### 2.5 Statistical analysis

All statistical analyses were done using R version 4.3.2. A Tukey Honest Significant Differences (TukeyHSD) test was used to provide confidence intervals for the various pairwise comparisons after an Analysis of Variance (ANOVA) was completed. The ggplot2 package version 3.5.1 was used to create all the plots, and multcompLetters4() from the multcompView package version 0.1.10 was used to add the letters of significance.

## 3 Results

### 3.1 PGP activities under drought stress

#### 3.1.1 Bacterial growth

**Figure 1:**
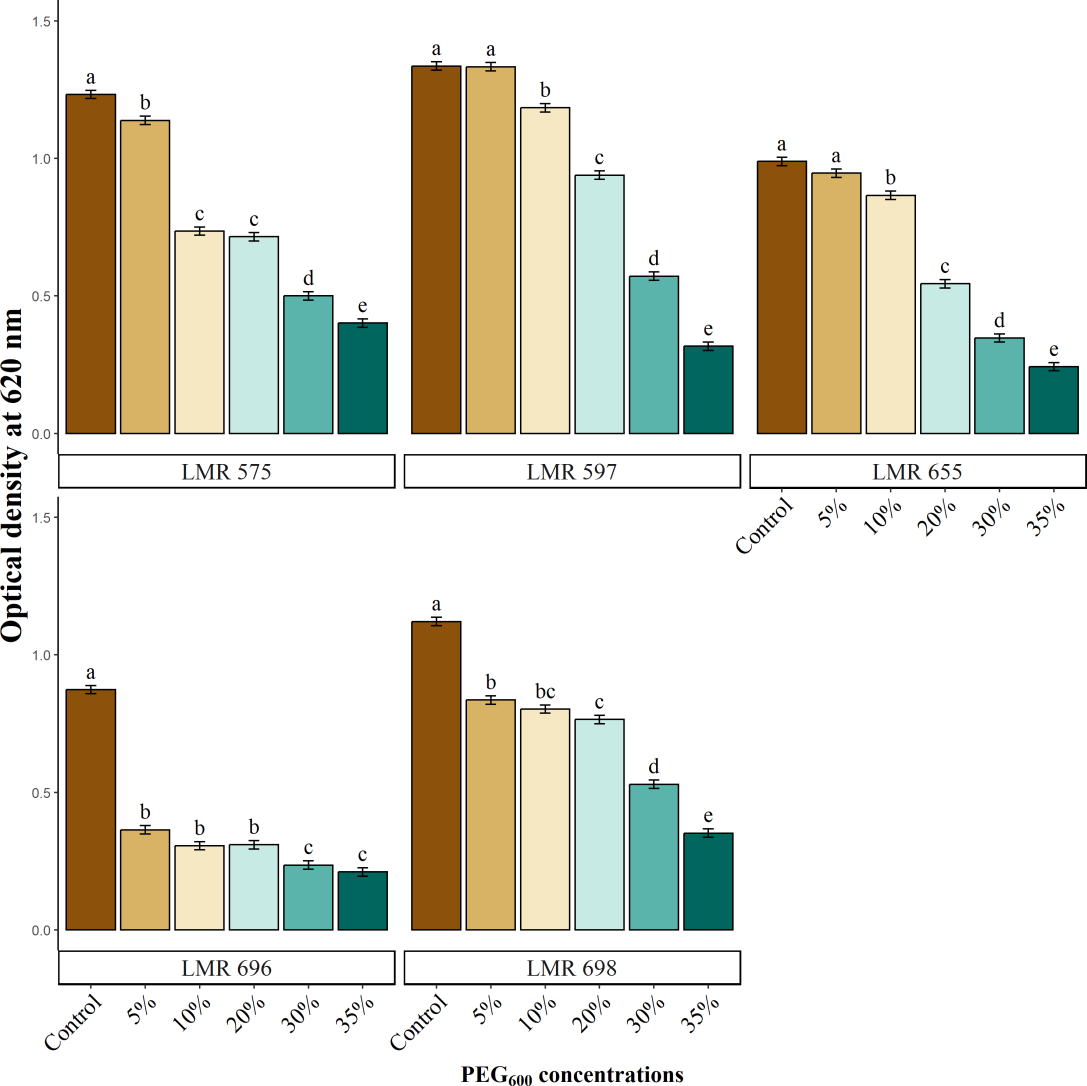
Effect of osmotic stress (PEG_6000_ concentrations) on growth of *R. laguerreae* strains LMR575, LMR597, and LMR655, *Enterobacter aerogenes* LMR696, and *Bacillus* sp. LMR698. Different letters represent significant statistical differences using Tukey-HSD tests at (p < 0.05).

Bacterial growth was assessed simultaneously with the measurement of auxin production. The results presented in **Figure 1** revealed the negative effect of water deficiency on bacterial growth, which showed a graduate decrease as PEG_6000_ concentrations increased. *R. laguerreae* LMR575, LMR597, and *Bacillus* sp. LMR 698, strains exhibited moderate growth compared the control, at a concentration of 30% maintaining an optical density above 0.5 at 620 nm. In contrast, *R. laguerreae* LMR 655 was able to tolerate up at 20% PEG_6000_. Meanwhile, *E. aerogenes* LMR 696 was highly sensitive to osmotic stress. Notably, all strains were found to be vulnerable to the high osmotic pressure applied by PEG_6000_ (35%).

#### 3.1.2 Auxin production

**Figure 2:**
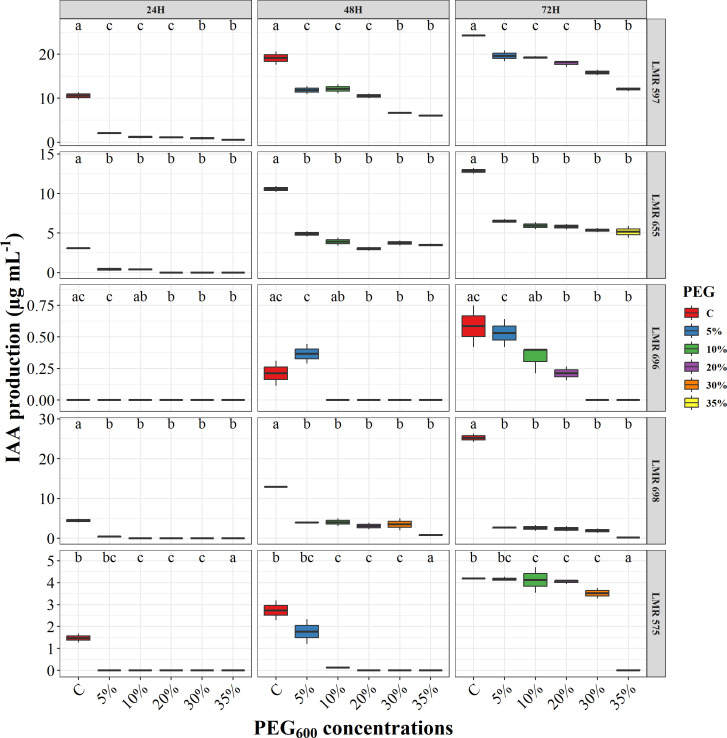
Effect of osmotic stress (PEG_6000_ concentrations) on auxin production of *R. laguerreae* strains LMR575, LMR597, and LMR655, *Enterobacter aerogenes* LMR696, and *Bacillus* sp LMR698. Different letters represent significant statistical differences using Tukey-HSD tests at (p < 0.05).

The maximum amount of auxin produced by bacteria in YEM medium supplemented with 50 μg mL^-1^ of L-tryptophan, was reached after 72 hours of incubation (**Figure 2**). The bacterial strains exhibited a greater capacity to produce auxin under normal conditions compared to stressful ones. For *R.laguerreae* LMR 597, the auxin production dropped from 24.22 µg/ml in normal conditions to 12.03 µg mL^-1^ at PEG_6000_ (35%). In contrast, auxin production by *Bacillus* sp. LMR 698 and *R. laguerreae* LMR 655 was only observed under normal conditions, with concentrations of 25.24 and 12.87 µg mL^-1^, respectively. Additionally, *R. laguerreae* LMR 575 exhibited the highest auxin synthesis after 72 hours of incubation, while *E. aerogenes* LMR 696 showed a gradual decrease in auxin synthesis as the PEG concentration increased. It is noteworthy, that except for *R. laguerreae* LMR 597, the activity of all strains, was significantly inhibited at PEG_6000_ (35%), with auxin synthesis falling below 5 µg mL^-1^.

#### 3.1.3 Siderophores production

**Figure 3:**
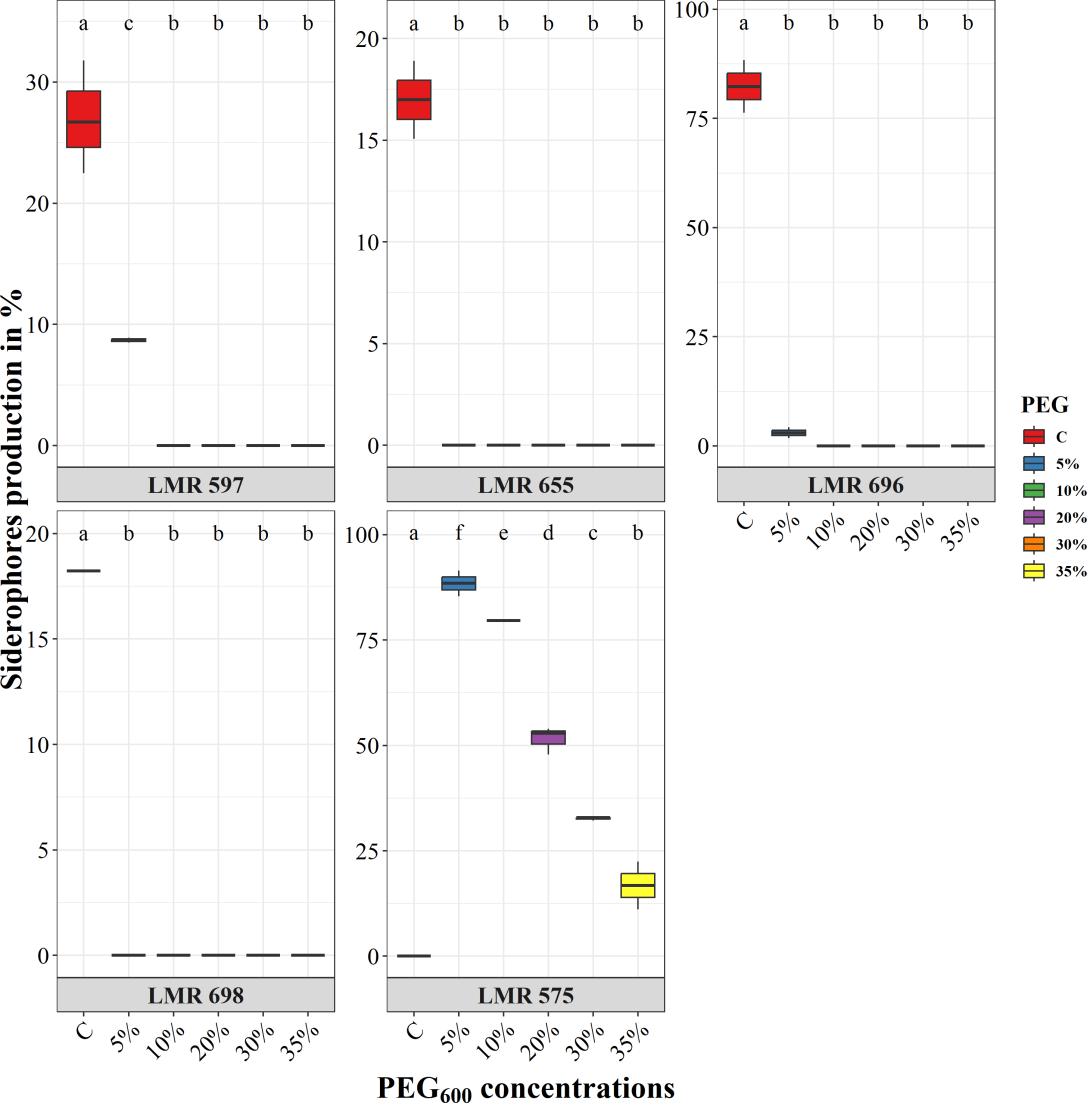
Effect of osmotic stress (PEG_6000_ concentrations) on siderophore production of *R. laguerreae* strains LMR575, LMR597, and LMR655, *Enterobacter aerogenes* LMR696, and *Bacillus* sp LMR698. Different letters represent significant statistical differences using Tukey-HSD tests at (p < 0.05).

Under normal conditions, *E.aerogenes* LMR 696 exhibited the highest level of siderophore production of 82%, followed by *R. laguerreae* LMR 597 with 27%, *Bacillus* sp. LMR 698 with 18%, and finally *R. laguerreae* LMR 655 with 17%. However, these strains showed no siderophore activity under varying water potential levels. Conversely, *R. laguerreae* LMR 575 showed a high level of siderophores production up to 88% at 5% PEG_6000_, but this production declined significantly with the increase in PEG_6000_ concentrations, reaching 16.78% at 35% of PEG_6000_ (**Figure 3**).

#### 3.1.4 Inorganic phosphate solubilization

**Figure 4:**
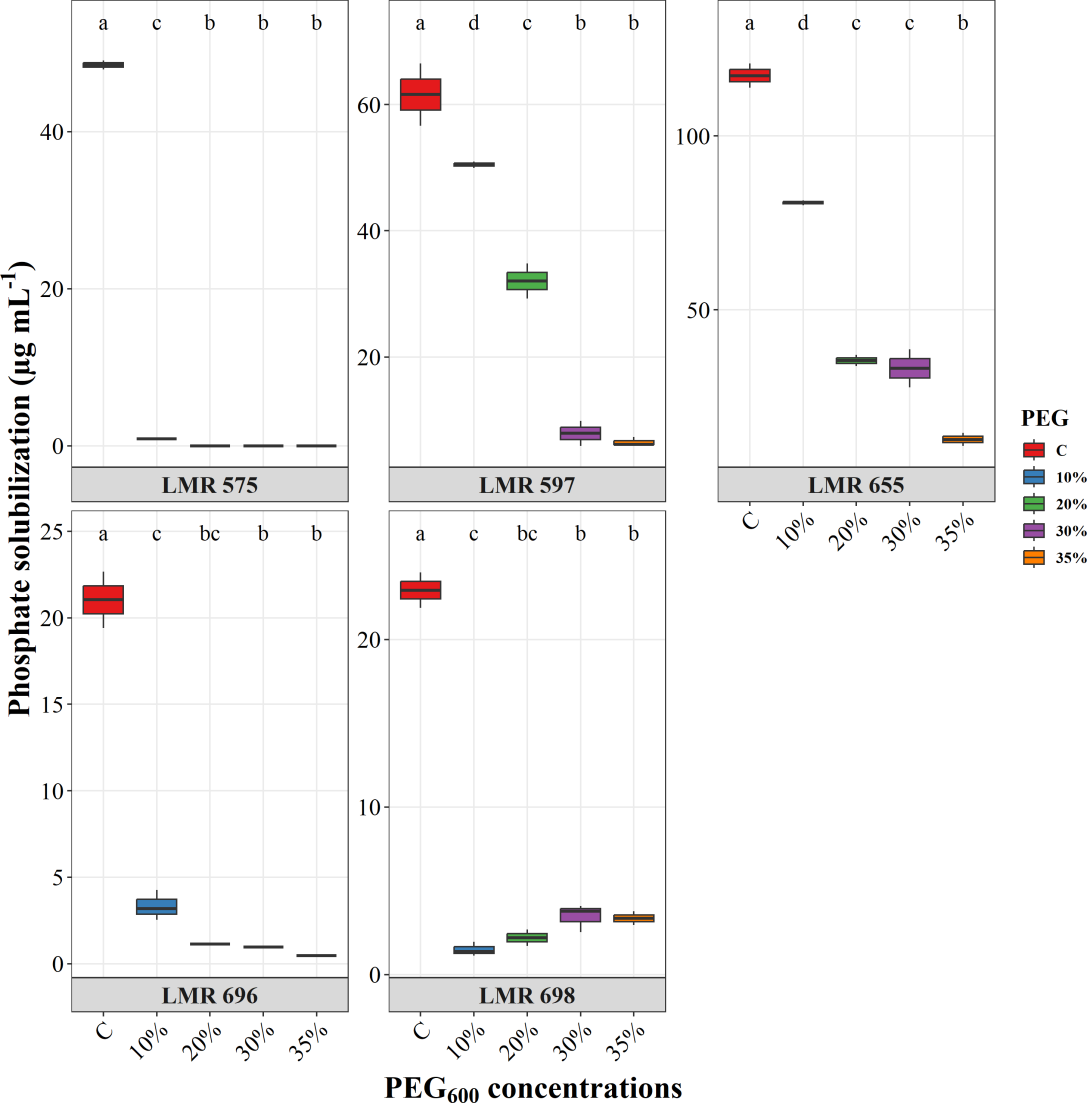
Effect of osmotic stress (PEG_6000_ concentrations) on phosphate solubilization of *R. laguerreae* strains LMR575, LMR597, and LMR655, *Enterobacter aerogenes* LMR696, and *Bacillus* sp. LMR698. Different letters represent significant statistical differences using Tukey-HSD tests at (p < 0.05).

All the bacterial strains tested demonstrated the ability to solubilize inorganic phosphate (**Figure 4**). The highest level of production was recorded by *R. laguerreae* LMR 655, which produced 113.85 µg mL^-1^. In comparison, *R. laguerreae* LMR 575 and LMR 597 showed moderate production levels of 48.5 and 61.57 µg mL^-1^, respectively. Conversely, *E.aerogenes* LMR 696 and *Bacillus* sp. LMR 698 exhibited lower solubilization rates, producing 22.5 and 20.5 µg mL^-1^, respectively. The ability of the studied strains to solubilize phosphate was significantly diminished compared to the control, and this decrease was correlated with increasing PEG concentrations. While *R. laguerreae* LMR 597 and LMR 655 could produce PO_4_²^-^ at a lower level of 30 µg mL^-1^ at 20% and 30% PEG_6000_, respectively. In contrast, *R. laguerreae* LMR 575, *E. aerogenes* LMR 696, and *Bacillus* sp. LMR 698 showed no phosphate solubilization capacity starting at 10% PEG_6000_.

### 3.2 Symbiotic host-range of *R. laguerreae* strains

The studied *R. laguerreae* strains, LMR 575, LMR 597, and LMR 655, were individually inoculated into *L. culinaris*, *P. sativum*, *V. faba*, and *Phaseolus vulgaris* seeds to determine their host range. The results indicated that all strains were capable of nodulating their host plant: *L. culinaris*. *R. laguerreae* LMR 597 and LMR 655 strains were also able to establish symbiosis with *P. sativum* and *V. faba* while no strain was able to form nodules in *P. vulgaris* (**Supplementary Figure S1**).

### 3.3 Effect of bacterial mixed inoculum on plants growth under osmotic stress

#### 3.3.1 *In-vitro* compatibility test

The *in-vitro* compatibility test revealed that there are no antagonistic interactions between the selected strains.

#### 3.3.2 Nodules number

Under non-stress conditions with inoculation, *P. sativum* and *V. faba* demonstrated high nodulation, with *P. sativum* producing over 80 nodules per plant and *V. faba* yielding an average of 67 nodules with minimal variation (SD = 0.52), indicating consistent nodule formation. In contrast, both species showed no nodulation under non-inoculated or KNO_3_-supplemented treatments, regardless of stress conditions. Under stress conditions, however, inoculated plants of *P. sativum* and *V. faba* still formed nodules, though in reduced numbers (22.67 and 41.33 nodules per plant, with SD values of 0.90 and 0.45 respectively) (**Table 1**).

**Table 1:** Nodules number in *Pisum sativum* and *Vicia faba* under different PEG treatments.

Treatment	*Pisum sativum*		*Vicia faba*	
	Nodules number	SD	Nodules number	SD
Non stressed + KNO_3_	0	0	0	0
Non stressed inoculated	> 80	–	67	0.52
Non stressed non inoculated	0	0	0	0
Stressed + KNO_3_	0	0	0	0
Stressed inoculated	22.67	0.90	41.33	0.45
Stressed non inoculated	0	0	0	0

Nodule number represents the average number of three replicates; SD is the standard deviation for each treatment.

#### 3.3.3 Shoot and root length

**Figure 5:**
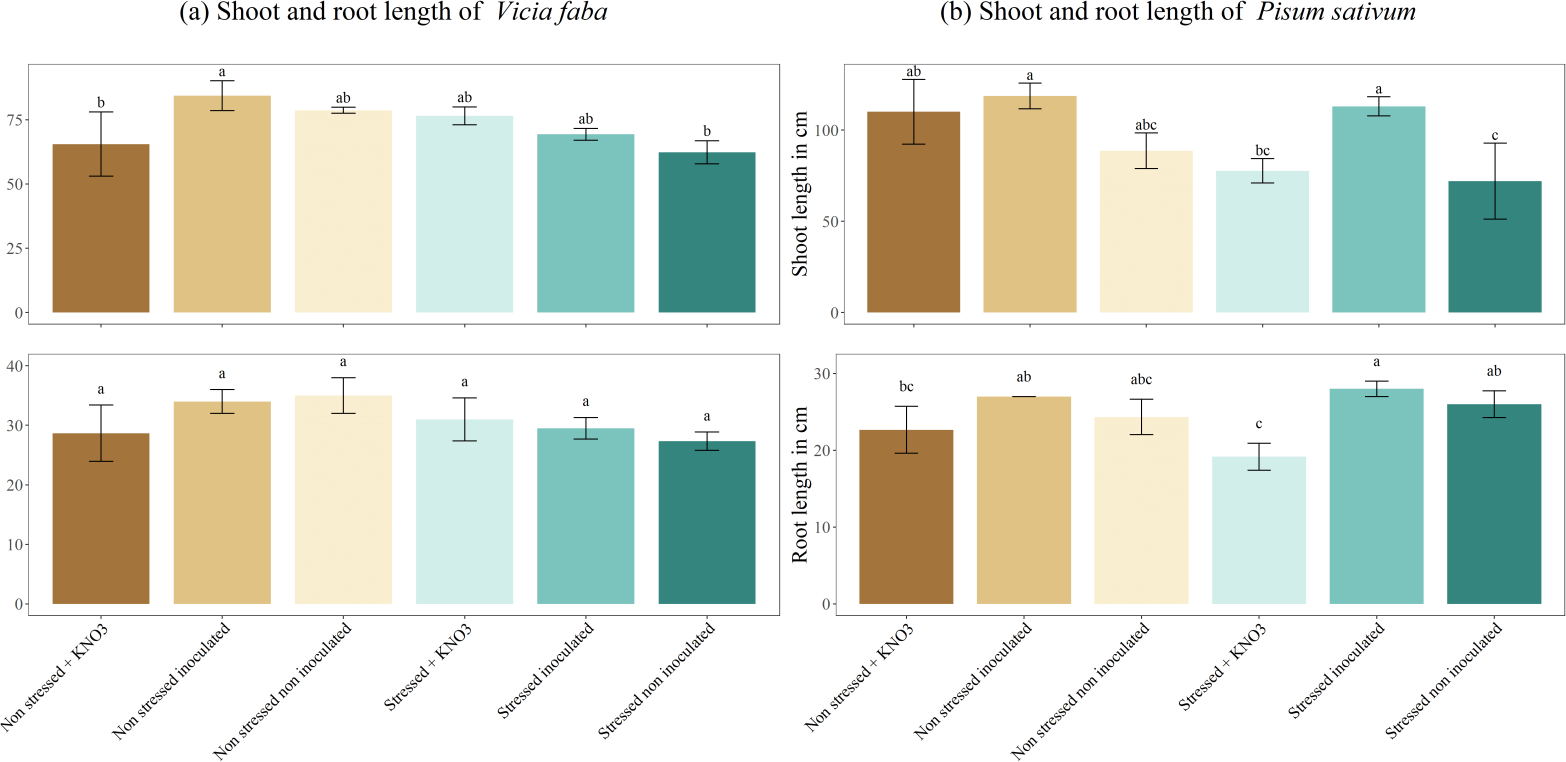
Shoot and root length under different treatments for two leguminous species, **(A)** *Vicia faba* and **(B)** *Pisum sativum*. Different letters represent significant statistical differences using Tukey-HSD tests at (p < 0.05).

As shown in **Figure 5A, B**, *V. faba* and *P. sativum* exhibited distinct responses in root and shoot length under various treatments. In *V. faba*, the non-stressed, inoculated plants showed the highest shoot length (84.33 cm), which was significantly greater than the non-stressed plants supplemented with KNO_3-_ and the stressed, non-inoculated plants, both of which did not differ significantly from the other treatments. Root length in *V. faba* remained relatively consistent across all treatments. In *P. sativum*, root length was significantly higher in stressed, inoculated plants (28 cm) compared to the stressed and non-stressed plants treated with KNO_3-_ (19 cm and 22 cm, respectively). The shoot length of stressed, non-inoculated *P. sativum* plants was notably lower (72 cm), differing significantly from stressed, inoculated plants and non-stressed plants supplemented with KNO_3-_ or inoculated, which all showed higher shoot lengths averaging around 113 cm.

#### 3.3.4 Chlorophyll

**Figure 6:**
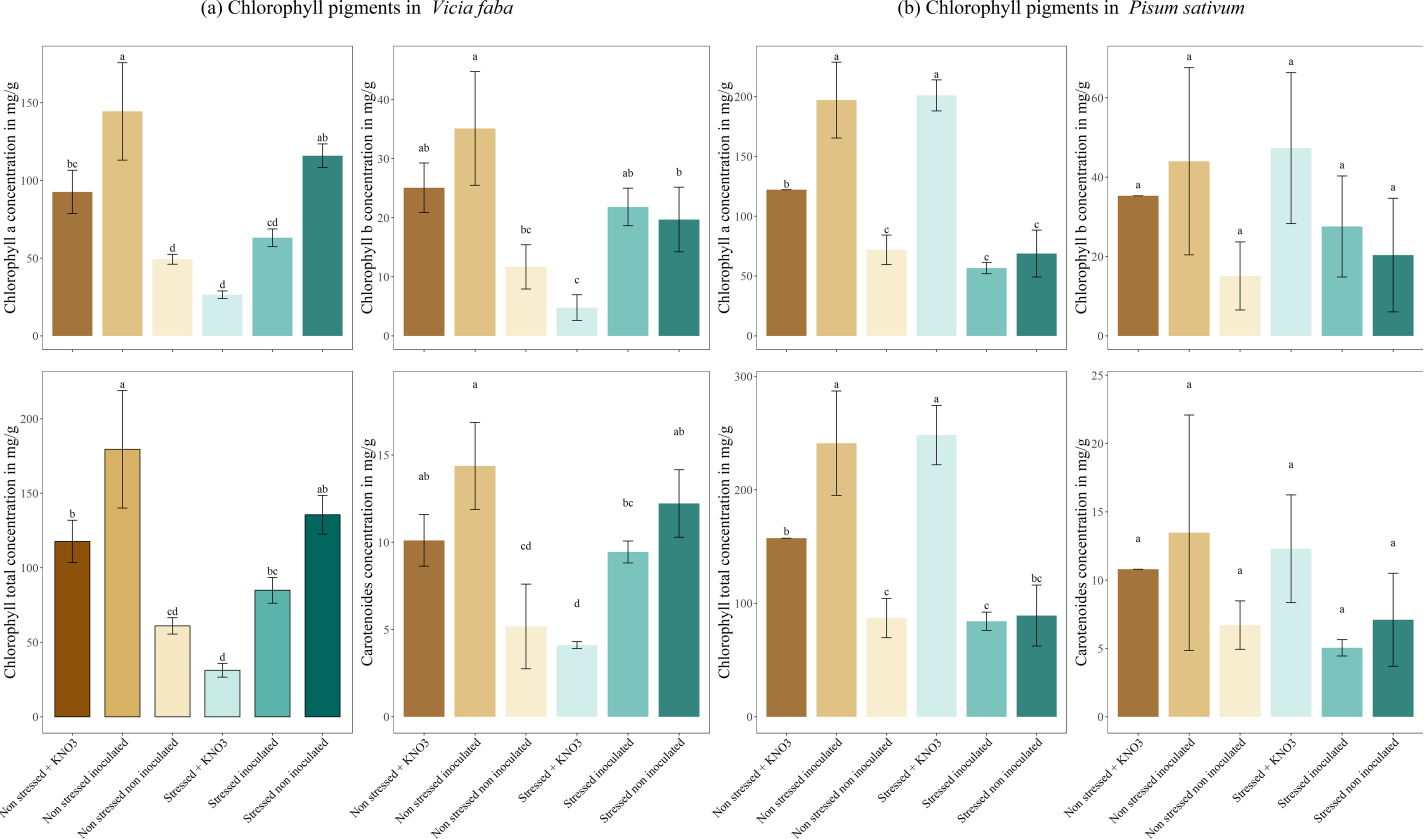
Production of photosynthetic pigments under different treatments for two leguminous species, **(A)** *Vicia faba* and **(B)** *Pisum sativum*. Different letters represent significant statistical differences using Tukey-HSD tests at (p < 0.05).

The chlorophyll and carotenoid content in *V. faba* and *P. sativum* demonstrated a clear response to inoculation and stress treatments. In both species, the non-stressed, inoculated plants exhibited the highest concentrations of chlorophyll (a), chlorophyll (b), total chlorophyll, and carotenoids, significantly surpassing non-inoculated and KNO_3-_supplemented plants. Specifically, non-stressed inoculated plants had a chlorophyll (a) concentration of 144.39 mg g^-1^, which was significantly higher than both non-inoculated and KNO_3-_supplemented plants. Regarding chlorophyll (b), inoculated plants under non-stressed conditions showed a concentration of 35.12 mg g^-1^, again surpassing non-inoculated and stressed non-inoculated plants. Under drought stress, inoculation mitigated the reduction in chlorophyll and carotenoid content more effectively than KNO_3-_ supplementation, with stressed inoculated plants in *V. faba* maintaining higher chlorophyll (a) and total chlorophyll levels (**Figure 6A**). In *P. sativum*, stressed inoculated plants similarly retained relatively higher chlorophyll (a) and carotenoid concentrations compared to KNO_3-_ treated plants. For total chlorophyll content, non-stressed inoculated plants displayed a significantly high concentration of 179.51 mg g^-1^, while under stress, inoculation increased total chlorophyll concentration to 84.90 mg g^-1^ compared to KNO_3-_supplemented plants. Interestingly, stressed non-inoculated plants retained relatively high chlorophyll (a) concentrations (115.85 mg g^-1^) compared to KNO_3-_treated plants (**Figure 6B**).

#### 3.3.5 Proline

**Figure 7:**
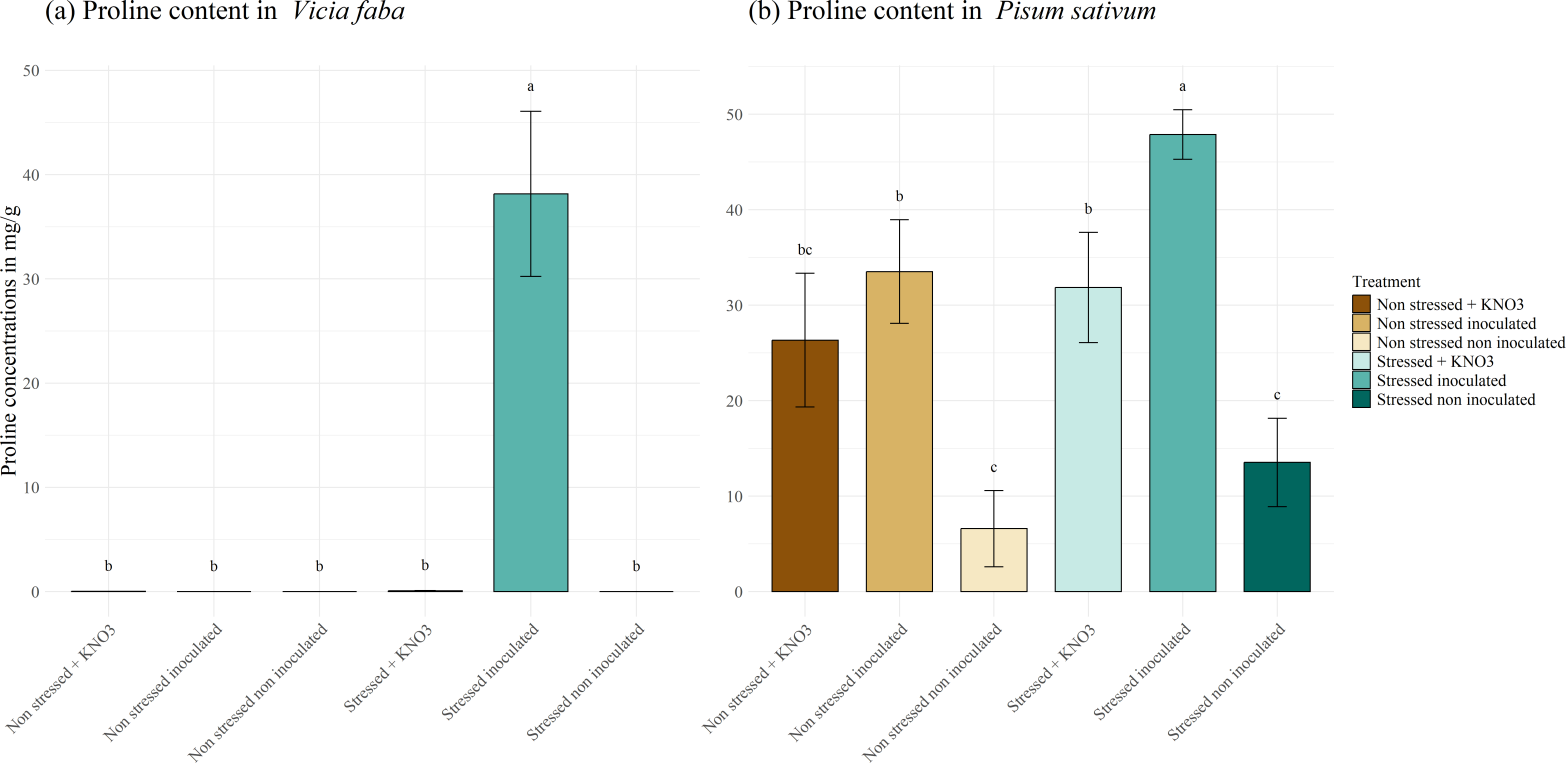
Proline production under different treatments for two leguminous species, **(A)** *Vicia faba* and **(B)** *Pisum sativum.* Different letters represent significant statistical differences using Tukey-HSD tests at (p < 0.05).

Proline production in *P. sativum* and *V. faba* under drought stress is shown in **Figure 7**. In both leguminous species, the level of proline produced under drought stress in plants treated with a mixed inoculum is higher with values of 48 and 38 mg g^-1^ for *P. sativum* and *V. faba* respectively, differing significantly with the other treatment. In *V. faba* (**Figure 7A**), no proline production was detected in plants under other treatment; Meanwhile the production of proline in *P. sativum* (**Figure 7B**) was not only related to the stress response but also to inoculation and KNO_3_ addition, where there was no significant difference in the amount of proline produced in stressed and non-stressed plant amended with KNO_3_ and non-stressed plant inoculated with the mixed inoculum. Whilst the lowest proline production was observed in non-stressed non-inoculated plants (6.5 mg g^-1^) and stressed non-inoculated plants (13.5 mg g^-1^).

#### 3.3.6 Anthocyanins

**Figure 8:**
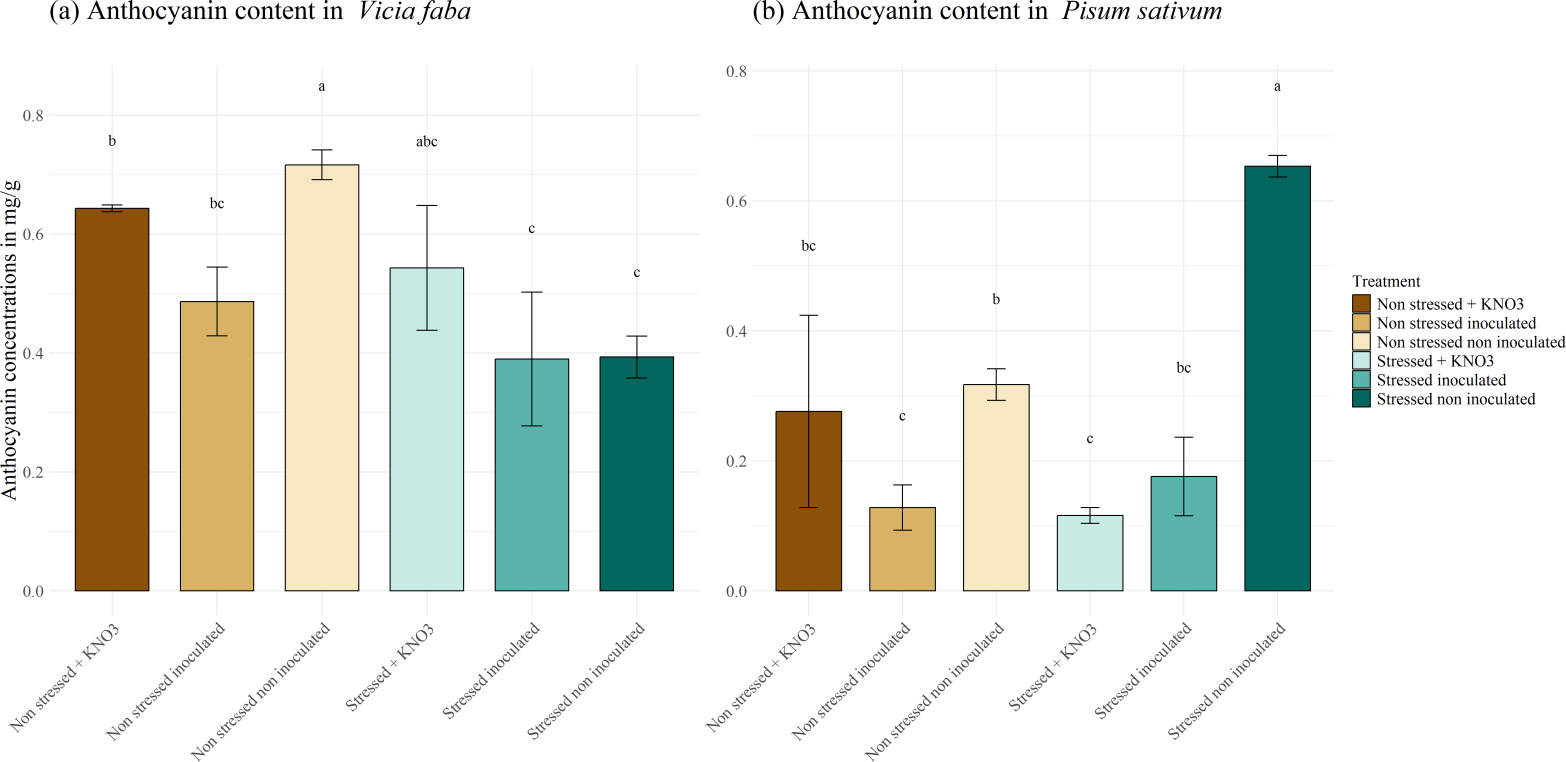
Anthocyanin biosynthesis under different treatments for two leguminous species, **(A)** *Vicia faba* and **(B)** *Pisum sativum*. Different letters represent significant statistical differences using Tukey-HSD tests at (p < 0.05).

Anthocyanins, pigments produced in response to stress, were also measured in our study (**Figure 8**). The results showed that concentrations did not exceed 1 mg g^-1^. In *V. faba*, anthocyanin synthesis did not appear to be associated with either stress response or the effect of inoculation (**Figure 8A**). Conversely, in *P. sativum*, the highest anthocyanin concentration was observed in the non-inoculated, stressed plants, reaching 0.65 mg g^-1^ which is significantly higher than in the inoculated plants and those supplemented with KNO_3_, where concentrations decreased to 0.176 and 0.116 mg g^-1^, respectively (**Figure 8B**).

#### 3.3.7 Soluble sugar

**Figure 9:**
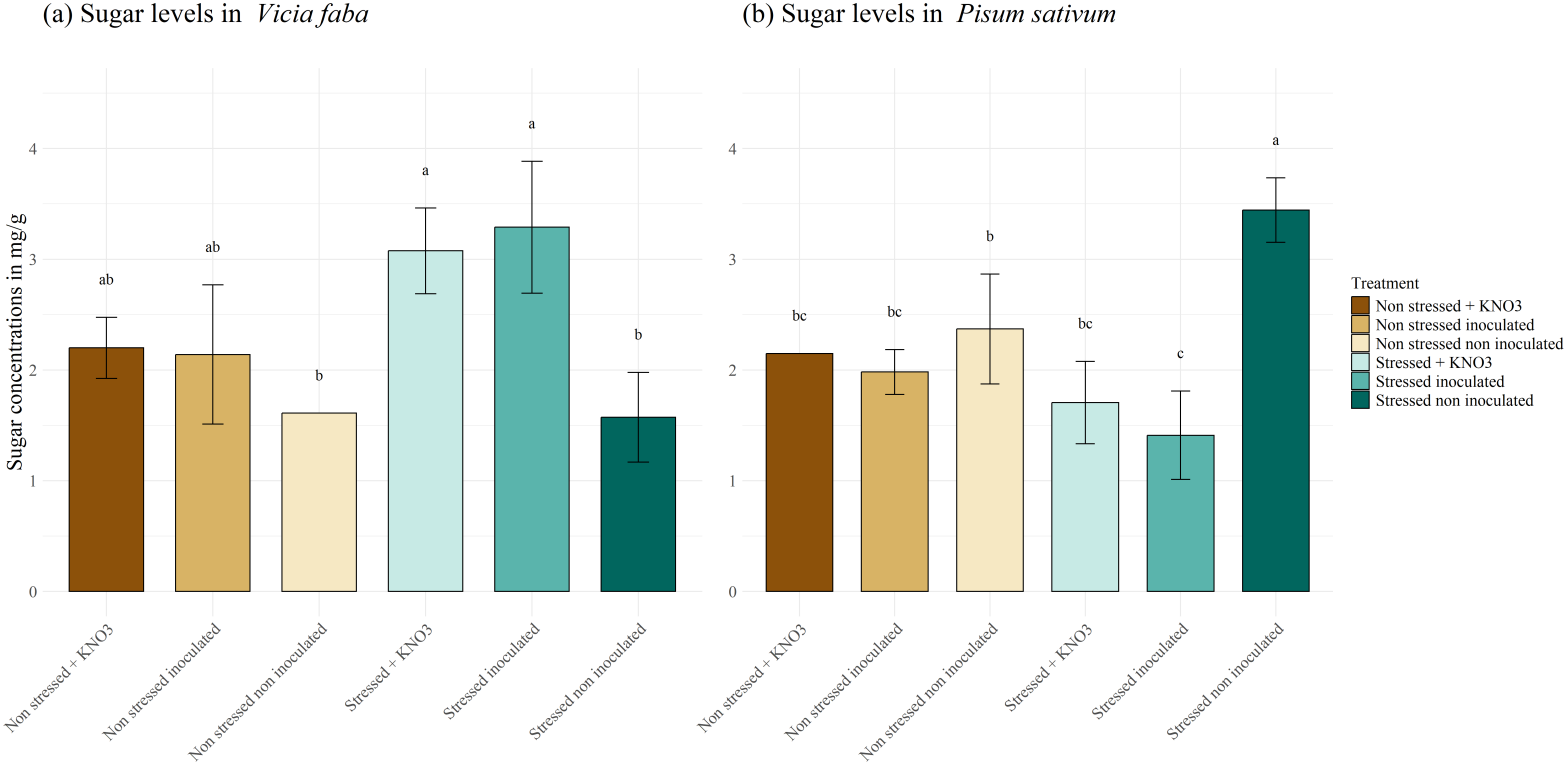
Total soluble sugar production under different treatments for two leguminous species, **(A)** *Vicia faba* and **(B)** *Pisum sativum.* Different letters represent significant statistical differences using Tukey-HSD tests at (p < 0.05).

The effect of inoculation on plant physiological responses varies between leguminous species. Examining the behavior of *V. faba* (**Figure 9A**), we observed a highly significant accumulation of soluble sugar in stressed inoculated plants, reaching 3.29 mg g^-1^, compared to 1.57 mg g^-1^ in stressed non-inoculated and 1.60 mg g^-1^ in the non-inoculated non stressed plants. Broadly speaking, both KNO_3_ treatment and inoculation helped maintain soluble sugar levels in stressed plants similarly to those under normal condition treated with a mixed inoculum and KNO_3_. Regarding *P. sativum* (**Figure 9B**), the highest significant concentration of soluble sugar was observed in the stressed non-inoculated plants reaching 3.44 mg g^-1^, compared to the other treatments. Simultaneously no significant difference was detected between the plants irrigated with KNO_3_ and those inoculated under the same stress condition, where the sugar concentration decreased to a range of 2.14 and 1.98 mg g^-1^ respectively.

#### 3.3.8 Proteins

**Figure 10:**
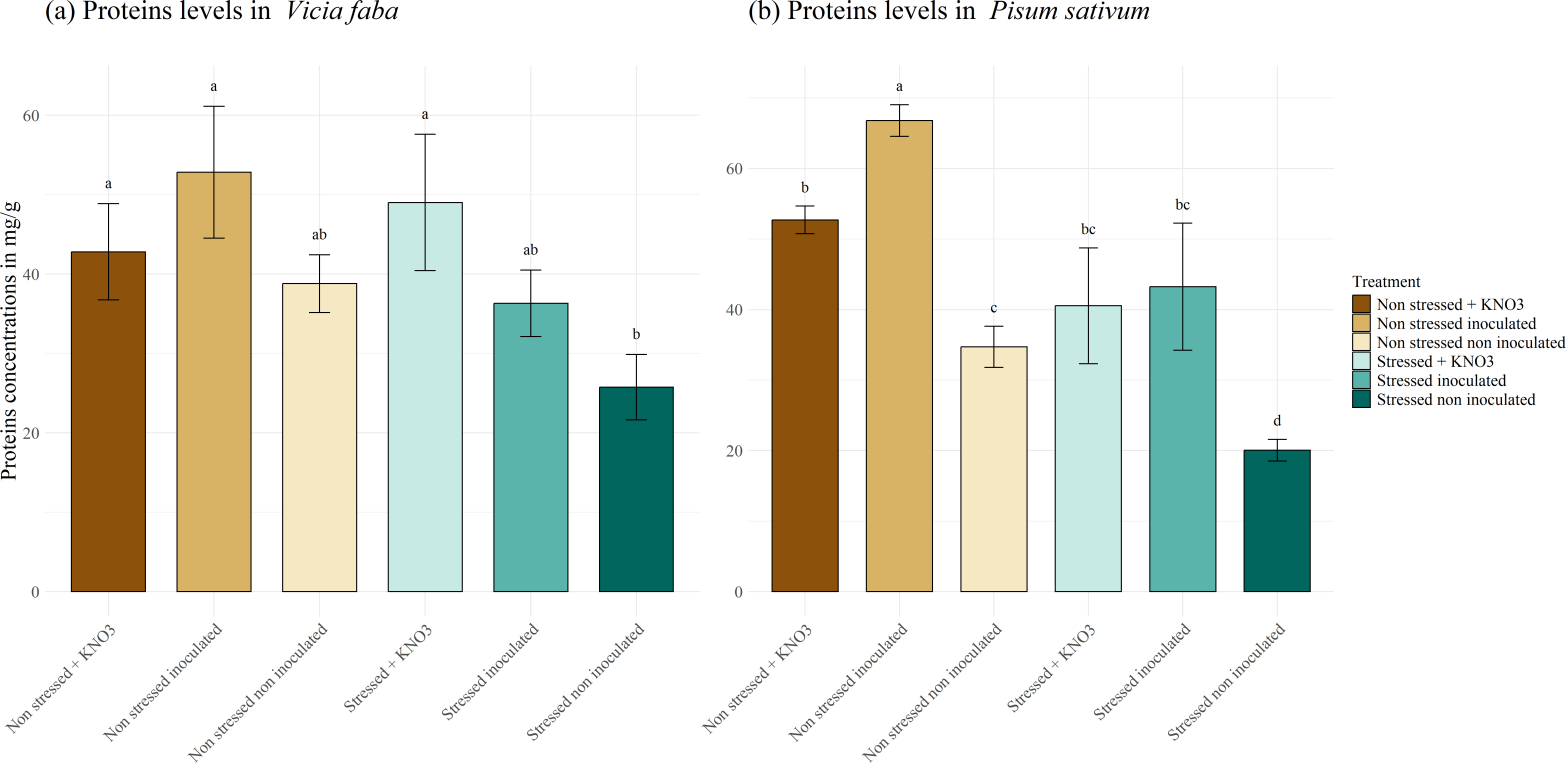
Proteins production under different treatments for two leguminous species, **(A)** *Vicia faba* and **(B)** *Pisum sativum.* Different letters represent significant statistical differences using Tukey-HSD tests at (p < 0.05).

The protein content in *V. faba* plants (**Figure 10A**) was impacted by the applied stress. The non-inoculated plants exhibited the lowest protein content of 25.74 mg g^-1^, which differed significantly from the plants amended with KNO_3,_ reaching a concentration of 49.03 mg g^-1^, whilst under normal conditions, no significant difference was observed among the applied treatments. Conversely, in *P. sativum* plants (**Figure 10B**), a significant effect of the inoculation was observed in the non-stressed plants achieving a concentration of 66.79 mg g^-1^, differing significantly from the plants supplemented with KNO_3_ (52.70 mg g^-1^) and the control (34.71 mg g^-1^). Meanwhile, the inoculated plants and those irrigated with KNO_3_ under drought stress displayed protein concentrations of 40.52 and 43.23 mg g^-1^ respectively, which were significantly higher than the stressed non-inoculated plants.

## 4 Discussion

This study builds upon previous research that focused on the isolation and identification of *Rhizobium laguerreae* as the primary nitrogen-fixing symbiont of *Lens culinaris* (lentil) across various regions in Morocco (Taha et al., 2018). Subsequently, the highest-performing strains were selected based on phenotypic screening (including pH, temperature, salinity, and osmotic stress tolerance), PGP potential, and symbiotic effectiveness. Settling on the final collection, which included three *R. laguerreae* strains LMR 575, LMR 597, and LMR 655 and two native PGPRs, *Bacillus* sp. LMR 698 and *Enterobacter aerogenes* LMR 696, these strains were then used in combinations for co-inoculation experiments with *L. culinaris* under stress conditions, generating promising results (Taha et al., 2022). These outcomes led us to question the ability of these bacteria to maintain their PGP traits under osmotic stress applied *in-vitro* using PEG_6000_.

Primarily, we assessed their growth and IAA production simultaneously, as several studies have been conducted in this area. The research conducted by Dhull et al., (2018) on *Rhizobium* strains isolated from the nodules of *Cymopsis tetragonoloba* in semi-arid regions of India revealed that all the strains tolerated up to 10% of PEG_6000_ with some able to grow at concentrations of 20% and 30%. Similarly, the isolated strain *Rhizobium* ‘QHCD11’ from (*Vicia faba* L.) in the arid region of China, that showed improved performance at 20% (Li et al., 2023). Moreover, out of a rhizobacteria collection isolated from a degraded soil of the north zone in Ethiopia, 10 isolates were able to tolerate an increased concentration of 40% PEG. However, bacteria that exhibit growth above 0.4 OD (Optical Density) are considered to be at the threshold for drought tolerance (Alikhani and Mohamadi, 2010). Likewise, our tested rhizobacteria could be considered drought-tolerant, with a high capacity to withstand water deficit, especially as *R. laguerreae* strains LMR575, LMR597 and *Bacillus* sp. LMR698 maintained stable population sizes at a PEG_6000_ concentration of 30%.

Previous studies have shown that bacteria tolerant to drought stress are able to display other PGP activities such as phytohormone production, nutrient availability in soil through phosphate solubilization and siderophore synthesis (El Attar et al., 2019; Zia et al., 2021, Taha et al., 2022). Corroborating our results, all the tested bacteria exhibited moderate performance in their PGP traits, which were influenced by the concentrations of PEG_6000_ applied, as obtained from the study of Sijilmassi et al. (2020). In their study, it was found that the production of IAA and phosphate solubilization ability of *Rhizobium* strains under drought stress decreased sharply with the severe application of osmotic stress. Furthermore, a study carried out on the behavior of three bacterial strains, *Proteus mirabilis* R2, *Pseudomonas b*al*earica* RF-2, and *Cronobacter sakazakii* RF-4, under drought stress, revealed that none of the strains exhibited PGP activity at the 10% PEG_6000_ stress level.

According to the results of our bacterial kinetics and their PGP potential, we observed that their activities are strongly dependent on their growth, both of which are negatively affected by elevated concentrations of PEG_6000_. This correlation can be explained by the disruption of the genetic regulatory mechanism, Quorum Sensing, which controls many bacterial functions, all of which are closely linked to bacterial population size (Williams, 2007).

This variability in PGP responses of each *R. laguerreae* strain was also reflected in their distinct symbiotic effectiveness with the tested leguminous species. However, our findings do not align with most studies, which isolated several species belonging to the genera *Rhizobium*, *Sinorhizobium, Mesorhizobium, Bradyrhizobium, Azorhizobium*, and *Agrobacterium* from the nodules of *Phaseolus vulgaris* (Martínez-Romero, 2003; El Attar et al., 2019). Furthermore, Flores-Félix et al. (2020) were the first to isolate *R. laguerreae* from *P. vulgaris* in a region where lentils were traditionally cultivated. These insights, and the unexpected loyalty of *R. laguerreae* LMR 575 to *Lens culinaris*, brought up several questions about the infection mechanism, the specificity of nodulation genes, and the whole-genome characteristics of these strains.

Combining the results mentioned above, the choice of mixed inoculum strains as inoculum was based on the idea of maximizing the benefits of the synergistic effects established by non-symbiotic PGPR and *Rhizobium*. In our case, this was a combination of *R. laguerreae* LMR 597 and LMR 655, which demonstrated the ability to produce auxin and solubilize inorganic phosphate, respectively, while establishing symbiosis with *P. sativum* and *V. faba*. Additionally, *E. aerogenes* LMR 696 was chosen for its siderophore production. This integration of symbiotic and non-symbiotic bacteria was outlined by various research works, which suggest that non-rhizobial strains could strengthen the effectiveness and efficiency of rhizobium with their plant host (Ben Gaied et al., 2024). These studies also highlight its potential as a promising biofertilizer that reduces the excessive use of chemical inputs, assists in mitigating challenging abiotic stresses such as drought through the induction of systemic tolerance (IST), and establishes beneficial interactions with leguminous plants (Yang et al., 2009; Khan et al., 2018).

In addition to its damaging effects on plant growth and nodule formation, drought stress also alters the physico-chemical properties of the soil, leading to infertility, susceptibility to disaggregation and deficiencies in essential minerals, such as nitrogen and phosphorus, that play a crucial role in plants physiological processes, including pigments synthesis, respiration and energy transfer (Wang et al., 2014; Dutta et al., 2018; Stavi et al., 2021). Thus, the application of symbiotic nitrogen fixers, phosphate solubilizers, IAA and siderophore producers, aimed to alleviate drought stress by promoting root elongation, regulating physiological and biochemical processes, enhancing nutrient availability in the soil, and improving biomass production (Deinum et al., 2016; Jabborova et al., 2021; Uzma et al., 2022).

The reduction in nodule numbers was the first clear sign of the severe stress applied to the inoculated plants. The absence of nodules in the non-inoculated plants and non-inoculated + KNO_3_ treatments in both leguminous species could be attributed to the absence of rhizobia in the soil or their inability to establish symbiosis with *P. sativum* and *V. faba* due to lower population sizes and effectiveness (Allito et al., 2021). Interestingly, the presence or absence of nodules did not correlate with the observed response in shoot and root growth of *V. faba*, nor did it indicate the effects of water deficiency and availability. Similarly to as shown by Ulzen (2018), where an increase in shoot dry weight following rhizobia inoculation in soybean was not noticed. This finding suggests that root morphology changes under water stress are species-specific (Mishra et al., 2020). On the other hand, the beneficial effect of the mixed inoculum was observed in the roots of *P. sativum*, where plants under stress exhibited the same performance as non-stressed, inoculated plants and the supplied with KNO_3_. This supports the role of the mixed inoculum in modulating root growth and architecture via IAA production, as observed in *Rhodobacter* sp*haeroides* KE149, a drought-tolerant, IAA-releasing bacterium that enhanced the growth of *Vigna angularis* var. *nipponensis* cv. *Arari* (Kang et al., 2020).

Chlorophyll (a) and (b) are the primary photosynthetic pigments in plants, directly facilitating photosynthesis, while carotenoid pigments are involved in dissipating excess excitation light energy (Khatun et al., 2021). Under drought stress, photosynthesis is one of the first physiological parameters to be affected, leading to a reduction in leaf water content and stomatal closure, which in turn causes photo-oxidation and degradation of chlorophyll pigments (Anjum et al., 2011; Yang et al., 2021). In *V. faba*, the mixed inoculum exhibited a promising result in enhancing chlorophyll content under normal condition as well as the application of KNO_3_, suggesting that application of this inoculum in normal irrigation conditions as biofertilizers rather than exogenous nitrogen could increase the absorption efficiency of water and nutrients in *V. faba* which are important for photosynthesis, which is an imperative process that improve yield trait (Mansour et al., 2021). Interestingly, stressed *V. faba* plants that were not inoculated produced higher amounts of photosynthetic pigments compared to those under stress, inoculated, and treated with KNO_3_. This observation aligns with findings from several experiments conducted under drought stress, where some cultivars of black gram (*Vigna mungo* L. Hepper) increased their chlorophyll content in response to water stress (Ashraf and Karim, 1991). Similarly, a chickpea variety (ILC482) exhibited higher chlorophyll (a) levels compared to other varieties during the vegetative stage (Mafakheri et al., 2010). For *P. sativum*, the observed non-significant differences in chlorophyll pigment production align with the findings of Lucas et al. (2023), who reported no chlorophyll degradation and an increase in all pigments in inoculated plants under stress. This variability in chlorophyll synthesis under osmotic stress can be attributed to the duration and severity of drought stress, along with differences in crop type, where some tolerant genotype of *Phaseolus vulgaris*, Glycine *max Merr*., *Hordeum vulgare*, and *Zea mays* can maintain their chlorophyll content in high level under drought stress (Monteoliva et al., 2021; Khatun et al., 2021). Chlorophyll synthesis in *P. sativum* and *V. faba* may be associated with several mechanisms that enable these drought-tolerant genotypes to preserve chlorophyll under such conditions, involving a combination of transcriptional regulation, enzymatic activity, and the stability of photosystem components. The study by Avramova et al. (2015) on maize leaf response to drought stress demonstrated a transcriptional up-regulation of the photosynthetic machinery throughout the entire leaf growth zone, with increased mRNA levels of photosynthesis-related transcripts and genes encoding enzymes involved in the synthesis of tetrapyrrole, which likely contributes to the maintenance or even enhancement of chlorophyll content. Additionally, it has been shown that certain components of the photosynthetic apparatus, particularly PSI and PSII, demonstrate notable resistance to water stress, further supporting the preservation of chlorophyll and the overall resilience of the photosynthetic system under water-deficient conditions (Nikolaeva et al., 2010).

Proline, soluble sugars, and proteins are important plant cellular osmolytes produced under stress conditions, they maintain normal cellular morphology, protect cell membrane stability, eliminate reactive oxygen species (ROS), and enhance plant adaptation (Etesami and Maheshwari, 2018). When plants are subjected to drought stress, these metabolites accumulate to reduce osmotic pressure (Ghosh et al., 2021; Jing et al., 2023). For proline activity, both plant species (*P.sativum* and *V.faba*) accumulated high concentrations of this osmolyte under stress when inoculated with the mixed inoculum. This finding aligns with results from two separate studies on maize cultivated under water deficiency, where the application of *Pseudomonas putida* strain GAP-P45 and *Bacillus thuringiensis* enhanced proline accumulation (Sandhya et al., 2010; Armada et al., 2014). Similarly, *Paenibacillus polymyxa* promoted proline production in tomato cultivars (Ghosh et al., 2019), and *Mesorhizobium ciceri* spp. increased proline accumulation in chickpea under drought stress (Abdela et al., 2020). Co-inoculation with *R. leguminosarum* and *P. putida* was also associated with proline and soluble sugar accumulation compared to the control (Mansour et al., 2021), and a mixed inoculum applied to maize under stress significantly enhanced proline and soluble sugar levels in the seedlings (Wang et al., 2018). Likewise, inoculated *V. faba* plants under stress showed increased accumulation of proline, soluble sugars, and protein. However, *P. sativum* plants exhibited high accumulation of only protein and proline, with no significant increase in soluble sugars under stress. These differences in osmolyte accumulation responses constitute a debate among researchers. Some consider that higher proline accumulation is associated with less-tolerant species, while others have reported that increased proline concentrations are a sign of greater drought tolerance mechanisms (Arteaga et al., 2020).

Globally, the distinction between stress responses and stress tolerance is often blurred, leading to misunderstandings in the explanation and interpretation of results related to osmolyte production under stress. Not all responses are directly linked to tolerance, even though stress tolerance mechanisms are grounded in specific stress responses. The accumulation of osmolytes can be viewed either as a response mechanism or as part of the stress tolerance strategy, depending on the species and possibly the specific plant parts, such as shoots and roots.

## 5 Conclusion

Our study highlights that selecting strains based on their growth and PGP traits under drought stress is a crucial factor, alongside the choice of host plant. Interspecific interactions between the mixed inoculum and *P. sativum* and *V. faba* were evaluated through various biochemical parameters. Inoculation enhanced drought tolerance in *V. faba* through multiple mechanisms, while most responses in *P. sativum* were more closely related to the plant’s metabolic characteristics. Under normal conditions, this inoculum also showed promising results in boosting plant growth and yield. Our findings contribute to a deeper understanding of how combinations of symbiotic strains and non-rhizobia influence leguminous symbiosis and drought response, supporting the potential of inoculant applications as an effective strategy to combat drought, improve tolerance, and enhance yields in the context of climate change.

## Data Availability

The original contributions presented in the study are included in the article/**Supplementary Material**. Further inquiries can be directed to the corresponding authors.
