# Supplementary material for: Enhancing drought tolerance in Pisum sativum and Vicia faba through interspecific interactions with a mixed inoculum of Rhizobium laguerreae and non-host beneficial rhizobacteria
Source: Front Plant Sci. 2025 Feb 26;16:1528923. doi: 10.3389/fpls.2025.1528923 (PMC11898328; doi:10.3389/fpls.2025.1528923)
Supplement: Supplementary file 1 [file DataSheet1.pdf]

Supplementary Data

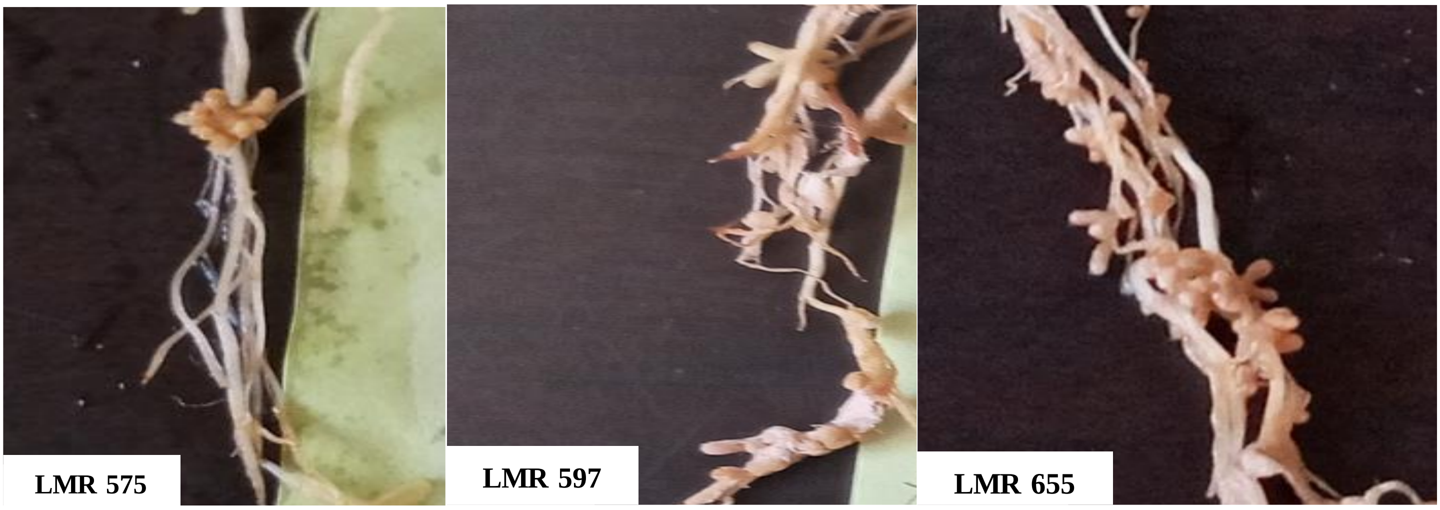

The nodules formed on the roots of *Lens culinaris* by different strains of *Rhizobium*.

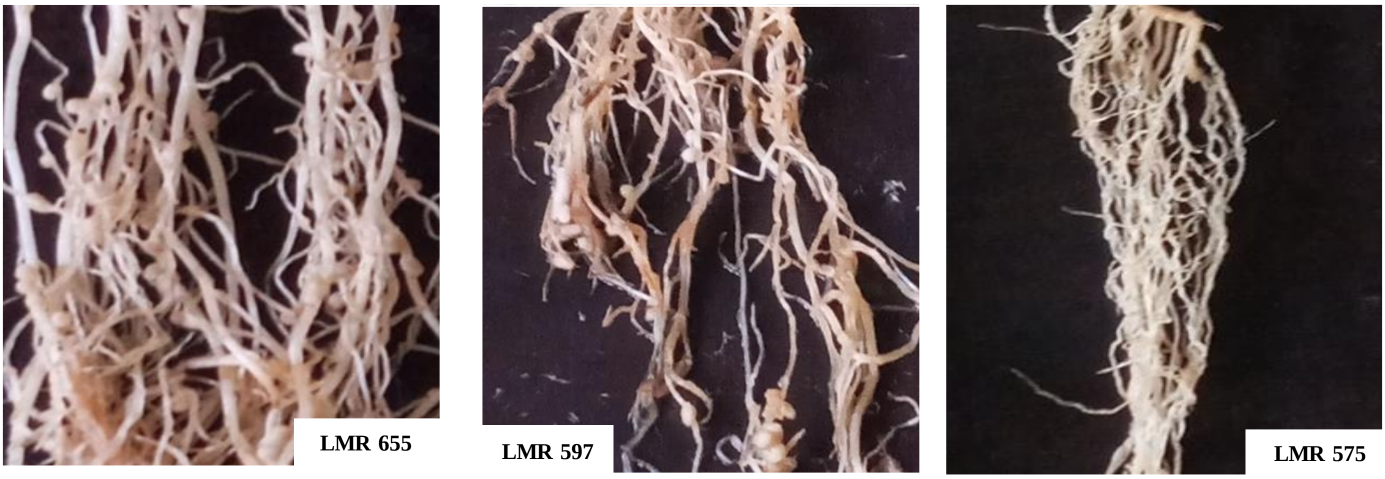

The nodules formed on the roots of *Pisum sativum* by different strains of *Rhizobium*

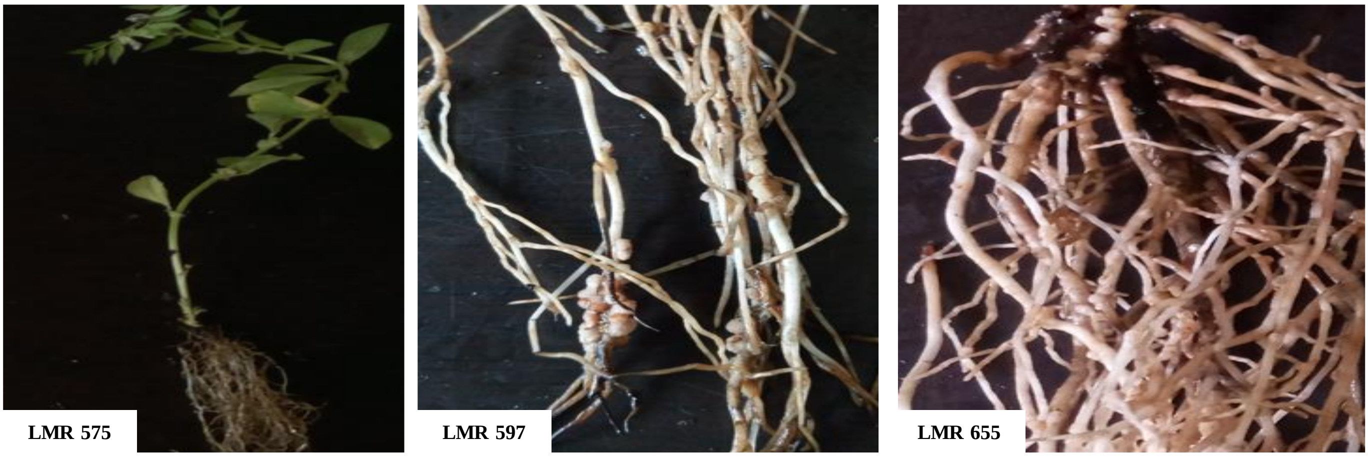

The nodules formed on the roots of *Vicia faba* by different strains of *Rhizobium*

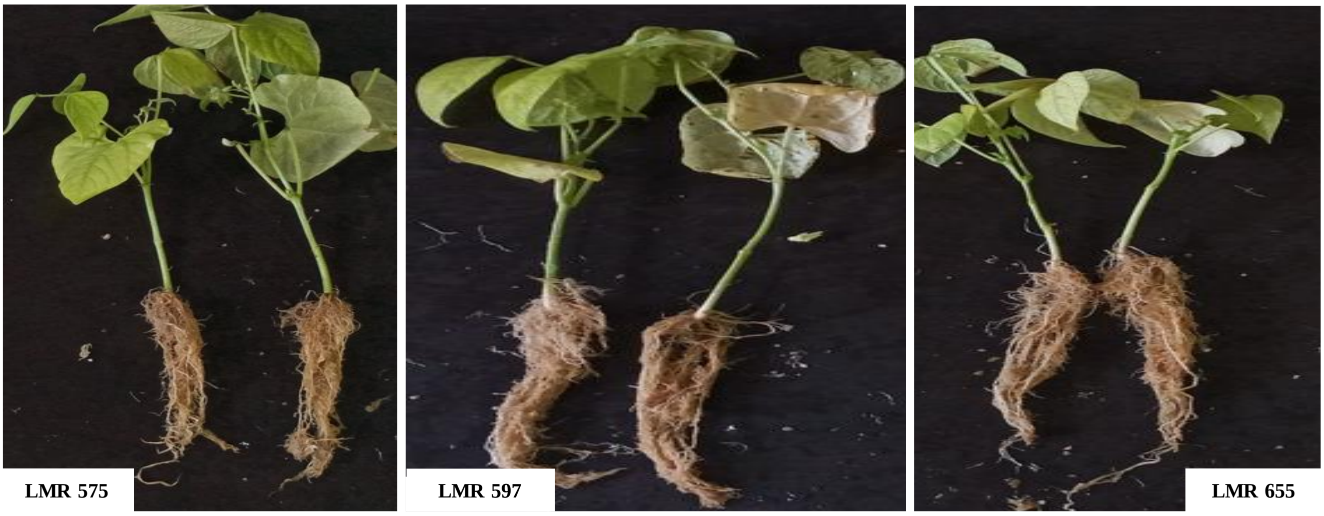

The nodules formed on the roots of *Phaseolus vulgaris* by different strains of *Rhizobium*
